# Postmenopausal Chinese-Singaporean Women Have a Higher Ratio of Visceral to Subcutaneous Adipose Tissue Volume than Caucasian Women of the Same Age and BMI

**DOI:** 10.3390/diagnostics11112127

**Published:** 2021-11-16

**Authors:** Maria Kalimeri, John J. Totman, Thomas Baum, Maximilian N. Diefenbach, Hans Hauner, Marcus R. Makowski, Karupppasamy Subburaj, David Cameron-Smith, Christiani Jeyakumar Henry, Dimitrios C. Karampinos, Daniela Junker

**Affiliations:** 1 Clinical Imaging Research Centre, Yong Loo Lin School of Medicine, National University of Singapore, Singapore 117599, Singapore; medv2358@nus.edu.sg (M.K.); jtotman@bournemouth.ac.uk (J.J.T.); 2 The Institute of Medical Imaging and Visualisation (IMIV), Bournemouth University, Bournemouth BH12 5BB, UK; 3 Department of Diagnostic and Interventional Neuroradiology, School of Medicine, Technical University of Munich, 81675 Munich, Germany; thomas.baum@tum.de; 4 Department of Diagnostic and Interventional Radiology, School of Medicine, Technical University of Munich, 81675 Munich, Germany; maximilian.diefenbach@tum.de (M.N.D.); marcus.makowski@tum.de (M.R.M.); dimitrios.karampinos@tum.de (D.C.K.); 5 Division of Infectious Diseases and Tropical Medicine, University Hospital, Ludwig Maximilian University of Munich, 80802 Munich, Germany; 6 Institute for Nutritional Medicine, School of Medicine, Technical University of Munich, 80992 Munich, Germany; hans.hauner@tum.de; 7 Else Kroener-Fresenius-Center of Nutritional Medicine, ZIEL Institute for Food and Health, Technical University of Munich, 85354 Freising, Germany; 8 Engineering Product Development Pillar, Singapore University of Technology and Design, Singapore 487372, Singapore; subburaj@sutd.edu.sg; 9 Singapore Institute for Clinical Sciences, Agency for Science, Technology and Research, Singapore 117609, Singapore; dcameron_smith@sics.a-star.edu.sg; 10 Riddet Institute, Massey University, Palmerston North 4442, New Zealand; 11 Liggins Institute, The University of Auckland, Auckland 1023, New Zealand; 12 Clinical Nutrition Research Centre, Singapore Institute for Food and Biotechnology Innovation, Agency for Science, Technology and Research, Singapore 117599, Singapore; jeya_henry@sics.a-star.edu.sg

**Keywords:** magnetic resonance imaging, visceral adipose tissue, subcutaneous adipose tissue, postmenopause, Caucasian, Asian

## Abstract

Central fat accumulation is a significant determinant of cardio-metabolic health risk, known to differ between ethnically distinct human populations. Despite evidence for preferential central adiposity in Asian populations, the proportional distribution between the subcutaneous and visceral compartments in Chinese postmenopausal women has not been thoroughly investigated. For this analysis, volumetrically quantified subcutaneous and visceral adipose tissue (SAT, VAT) in the pelvic and abdominal regions of postmenopausal Asian (Chinese-Singaporean) and Caucasian (German) women matched for age and Body Mass Index (BMI) was undertaken, to examine such differences between the two groups. Volumes were calculated from segmentations of magnetic resonance imaging datasets of the abdomen and pelvis. Despite SAT, VAT, and the corresponding total adipose tissue (TAT) being similar between the groups, VAT/SAT and VAT/TAT were higher in the Asian group (by 24.5% and 18.2%, respectively, each *p* = 0.02). Further, VAT/SAT and VAT/TAT were positively correlated with BMI in the Caucasian group only (*p* = 0.02 and *p* = 0.01, respectively). We concluded that VAT is proportionally higher in the non-obese Asian women, compared to the Caucasian women of matched age and BMI. This conclusion is in agreement with existing literature showing higher abdominal adiposity in Asian populations. Additionally, in the Asian group, BMI did not correlate with visceral adiposity on a significant level. Further analysis is required to examine the extent to which this increased VAT may impact cardio-metabolic health. There is, however, a need to emphasize healthy lifestyle behaviors in non-obese post-menopausal women of Chinese ancestry.

## 1. Introduction

Body fat distribution has been shown to correlate with the risk of various chronic diseases. Such diseases include metabolic syndrome and type 2 diabetes [1,2,3], cardiovascular disease [4,5], and certain cancers [6]. Moreover, a high ratio of visceral to subcutaneous abdominal fat is a specific risk factor for the development of atherosclerosis [5] and impaired glucose and lipid metabolism [7].

Fat distribution is affected by several factors, such as sex, age, and ethnicity. Men tend to store fat viscerally to a larger degree than women. With age, the amount of fat stored in the visceral compartments increases [8,9,10], although it has been suggested that the rate of fat accumulation decreases [11]. Ethnicity also appears to be a determinant of body fat distribution [12,13,14,15,16]. It has been shown that, after taking age, sex and anthropometric characteristics into account, Caucasians tend to have higher volumes of visceral fat (visceral adipose tissue, VAT) and subcutaneous fat (subcutaneous adipose tissue, SAT) than Africans and Inuit [17]. For a given body size, Asians have been shown to accumulate higher levels of VAT than other ethnic groups (Caucasian, Hispanic, and Caribbean of African descent) [18], but also overall adiposity differs among Asians from different geographic regions [19]. Premenopausal African-American women were found to have lower VAT volumes than Caucasian women with similar body mass index (BMI), while the SAT was not significantly different between the two groups [12]. A multi-site, multi-ethnic study showed significant differences in VAT volumes between different ethnic groups, with Asian groups having the highest levels of VAT for a given BMI [18]. Suggested reasons behind such findings are differences in body build and different rates of fat accumulation per compartment for different ethnic groups [13]. As a consequence, the prevalence of type 2 diabetes and metabolic syndrome is higher in Asian populations than among Caucasians, and Asians tend to develop these diseases at a younger age [20], while cardiovascular disease is also high in certain parts of Asia [21]. 

It has been suggested that after menopause women’s risk for diabetes and cardiovascular disease increases considerably, relative to the premenopausal period [22,23,24]. Even though the rate of fat accumulation is not affected by menopause [11], the menopausal transition is a period during which fat accumulation in women shifts from subcutaneous to visceral [25,26]. Thus, examining differences in truncal fat volume between groups of postmenopausal women from different racial groups adds information on identifying and quantifying disease risk in different populations. Although there have been studies comparing body fat distribution of Caucasian and Asian populations [27,28,29], studies focusing mainly on postmenopausal women are limited [30].

Body size and body fat content are most commonly described in terms of BMI. Calculated easily as the ratio of weight to height squared (kg/m^2^), it is ideal for large population studies. However, it has been recognized that the cut-off points used to define obesity need to be adjusted for different racial populations [31,32]. Additionally, the use of BMI shows low sensitivity in identifying individuals with a healthy weight but with high levels of body fat [33]. Thus, BMI is only a crude estimate of body size and is not an accurate tool for the assessment of disease risk [34]. Anthropometric measurements that consider abdominal fat distribution are waist circumference and the ratio of waist to hip circumference. In contrast, body fat content is estimated with methods such as skinfold thickness, bioelectrical impedance, and densitometry, but are also rather crude measures. Additionally, none of these methods offers a clear differentiation between VAT and SAT, which are known to have different health outcomes [35,36]. The distinction between VAT and SAT is made possible with the use of 3D imaging technologies, such as computed tomography (CT) and magnetic resonance imaging (MRI). MRI, in particular, does not expose the subjects to ionizing radiation and is thus the preferred method for adipose tissue imaging in healthy individuals, as well as for measurements repeated at multiple time points. To keep the scanning and post-processing time as low as possible, typically only a single slice of the abdomen is scanned, the L_4_-L_5_ level of the lumbar spine [37]. Measurements at that level correlate strongly with adipose tissue volumes of the whole abdomen [38], but this approach has certain drawbacks. It risks producing inaccurate results in non-homogeneous groups, as it has been reported that the maximum VAT area occurs at different levels relative to L_4_-L_5_ for people of different age, sex, or ethnicity [8]. Additionally, it has been shown to poorly predict VAT and SAT changes during weight loss [39]. In such cases, more accurate results are achieved by measuring the adipose tissue volume in a multi-slice or volumetric approach.

Thus, the purpose of this analysis was to volumetrically examine possible differences in the abdominal and pelvic fat accumulation of age- and BMI-matched Asian and Caucasian postmenopausal women cohorts using quantitative MR images. 

## 2. Materials and Methods

### 2.1. Subjects

The subjects examined in this analysis were recruited independently in two locations. The Asian cohort was recruited in Singapore, at the Clinical Nutrition Research Centre, Singapore Institute for Clinical Sciences. Recruitment took place through announcements on noticeboards in the National University of Singapore (NUS) campus and word of mouth, for a study on biomarkers for early detection of prediabetes (indexed in www.clinicaltrials.gov under NCT03309254, accessed on 1 June 2021). In total, 97 healthy, non-obese, postmenopausal Chinese-Singaporean women were recruited. The participants were between 55–70 years old (mean age 60.7 years) and their mean BMI was 22.9 (18–30 kg/m^2^). They were at least 5 years postmenopausal and in good general health, with no history of conditions such as osteoporosis, diabetes, arthritis, cancer, renal, or liver disease. Moreover, they did not smoke, consumed less than two units of alcohol daily, and, in the past 6 months, they had not been on medication such as glucocorticoids, hormone replacement therapy, and vitamin D supplements or calcitonin. The ethical committee of the National Healthcare Group, Singapore, approved the study and all participants provided written informed consent. 

For the Caucasian cohort, datasets fulfilling the inclusion criteria (female, postmenopausal defined as no menses for 12 or more consecutive months) were extracted from a database at the Institute of Diagnostic and Interventional Radiology, Klinikum Rechts der Isar, School of Medicine, Technical University of Munich. In total, 68 datasets from 43 women between 48 and 77 years old (mean age 59.1) and mean BMI 29.9 kg/m^2^ (18.1–43.5 kg/m^2^), were extracted. The database included pseudonymized datasets from several completed studies, among them of women from a wide range of BMI, in good general health with no history of diabetes. Exclusion criteria in all studies were a history of severe diseases (e.g., cancer, renal dialysis, stroke, inflammatory bowel disease) and surgeries within the last three months or acute physical limitations. All studies had been approved by the Institutional Review Board of the School of Medicine, Technical University of Munich, and written informed consent had been obtained from all participants. 

Two BMI- and age-matched groups resulting from the Asian and the Caucasian cohorts were created using MedCalc for Windows, version 19 (MedCalc Software, Ostend, Belgium), allowing for a maximum age and BMI difference of 3 years and 0.7 kg/m^2^, respectively, for each resulting pair. In Caucasian participants with multiple image datasets, only one dataset per subject was used. The resulting two groups consisted of 22 subjects each, resulting in a total of 44 subjects included in the analysis. 

### 2.2. Adipose Tissue Volume Measurements

All subjects underwent MR imaging of the abdomen and pelvis. For the Asian cohort, images were acquired at the Clinical Imaging Research Centre (CIRC), Yong Loo Lin School of Medicine, NUS, in a 3T Siemens Prisma scanner (Siemens Healthineers, Erlangen, Germany). Participants were scanned in the supine position and two stacks of axial two-echo Dixon images with 2 mm slice thickness were acquired, covering the abdomen and the pelvis. The acquisition time per stack was 1 min and 56 s. The Caucasian cohort underwent MRI scanning at the Department of Diagnostic and Interventional Radiology, Klinikum Rechts der Isar, School of Medicine, Technical University of Munich, in a 3T Philips Ingenia scanner (Philips Healthcare, Best, The Netherlands). Two stacks of axial two-echo Dixon 3D spoiled gradient-echo images with 5 mm slice thickness were acquired, covering the abdomen and the pelvis. The acquisition time for each two-echo Dixon sequence was 10.6 s and each scan was performed in a single breath-hold (Table 1).

### 2.3. Calculation of SAT/VAT Volumes

SAT and VAT volumes were extracted from the water- and fat- separated images of the two-echo Dixon scans, using a custom-built semi-automatic post-processing algorithm (http://doi.org/10.5281/zenodo.3545989 accessed on 1 June 2021) [40,41]. The area of interest was defined as the region from the liver dome to the middle of the femoral head. Segmentations resulting from the algorithm were adjusted slice-by-slice manually by one researcher per ethnic group (M.K. for the Singaporean group and D.J. for the Caucasian group), to exclude non-VAT compartments (e.g., intermuscular, around the spine) and thoracic fat from the quantification [42]. The two researchers initially agreed on a common approach in order to perform the manual corrections in the segmentations in consensus. The volume of each compartment was calculated as the total number of pixels multiplied by the voxel size (slice thickness × pixel width × pixel depth). The total adipose tissue (TAT) of the analyzed area was calculated as the sum of SAT and VAT [43].

### 2.4. Statistical Analysis

Statistical analysis was performed using SPSS for Windows, version 25.0 (IBM SPSS Statistics, SPSS Inc., Chicago, IL, USA). Normality was tested in each group separately, using the Shapiro–Wilk test. Between-group differences were examined by means of a *t*-test, adjusted for equal and non-equal variances as required, while for non-normally distributed parameters, between-group differences were examined using the Mann–Whitney non-parametric test. The relationships of adipose tissue volumes (absolute and relative) with age and BMI were examined using Pearson correlation and non-parametric Spearman rank correlation, as required. Pearson correlation coefficients were calculated for the relationships of SAT and TAT with BMI, and Spearman’s rho was calculated for the relationships of all fat measurements with age and also VAT, VAT/SAT, and VAT/TAT with BMI. Results were reported as statistically significant when *p* < 0.05.

## 3. Results

**Figure 1:**
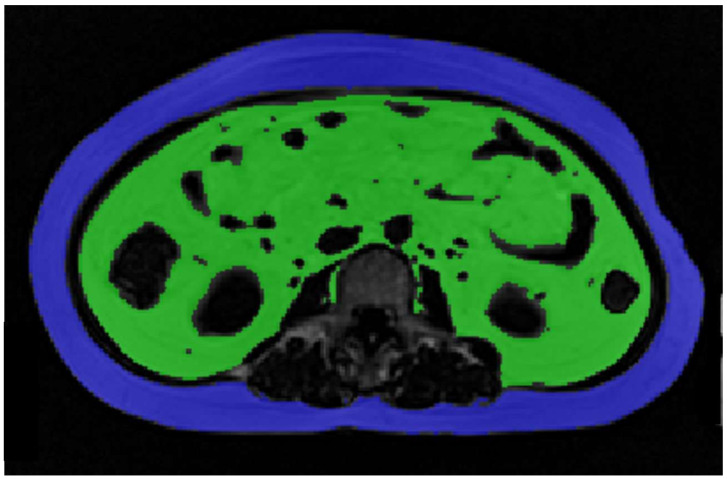
Slice at the level of the third lumbar vertebra, images acquired from the participant with unusually high levels of VAT (green color). SAT is shown in blue. Abbreviations: SAT: Subcutaneous adipose tissue, VAT: Visceral adipose tissue.

The measured characteristics of the subjects of each age- and BMI-matched group and the *p*-values of the *t*-tests and Mann–Whitney tests are shown in Table 2. Age in both groups and VAT, VAT/SAT, and VAT/TAT in the Asian group were not normally distributed. The reason for the non-normality of fat measurements in the Asian group is the very high VAT volume (higher than 3 standard deviations from the mean) measured in one participant, which distorted the distributions of these variables. This high value does not appear to be due to image artifacts or quantification error, and thus we decided not to exclude the participant containing the outlier measurement. An axial slice from the middle of L3 of the images acquired from this participant is shown in Figure 1. 

**Figure 2:**
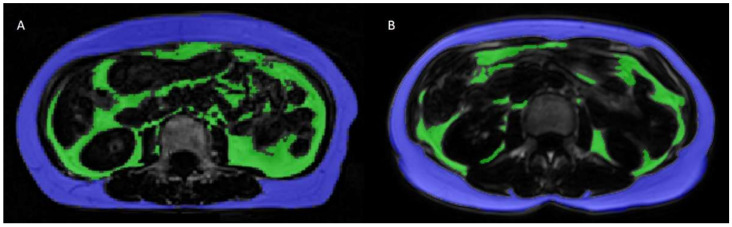
Side-by-side images showing a slice from images of age- and BMI-matched participants from the Asian cohort (A, BMI = 18.8 kg/m^2^) and the Caucasian cohort (B, BMI = 18.1 kg/m^2^) at the level of L3. Note the difference in VAT volume with higher VAT in participant A despite the similar BMI. VAT: green, SAT: blue. Abbreviations: BMI: Body Mass Index; SAT: Subcutaneous adipose tissue; VAT: Visceral adipose tissue.

As expected from the study design, age and BMI were not significantly different between the Asian and Caucasian groups. There were 10 women with a BMI ≥ 25 kg/m^2^ in the Caucasian group and 11 in the Asian group, the remainder of the two groups exhibited a BMI < 25 kg/m^2^. The Caucasian group was significantly taller than the Asian group. The length of the region analyzed (from the top of the liver to the middle of the femoral head) was marginally longer in the Asian group (*p* > 0.05) and the ratio between the length of the analyzed region and body height was significantly higher in the Asian group. Absolute values of visceral fat and the corresponding subcutaneous and total fat (VAT, SAT, TAT) were not significantly different between the two groups. The VAT/SAT and VAT/TAT ratios in the analyzed region were higher in the Asian cohort (Table 2). Figure 2 shows a side-by-side comparison of two age- and BMI-matched subjects. The Asian subject, shown in this figure, had a slightly higher BMI (18.8 kg/m^2^) than the Caucasian subject (18.1 kg/m^2^) and a much higher VAT volume.

**Figure 3:**
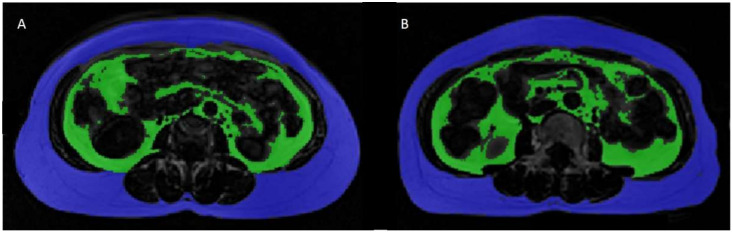
Images from two participants from the Asian cohort with similar VAT/SAT ratios (VAT/SAT = 0.33) and different BMI (**A**: BMI = 24.6 kg/m^2^, **B**: BMI = 22.1 kg/m^2^). Abbreviations: BMI: Body mass index; SAT: Subcutaneous adipose tissue; VAT: Visceral adipose tissue.

**Figure 4:**
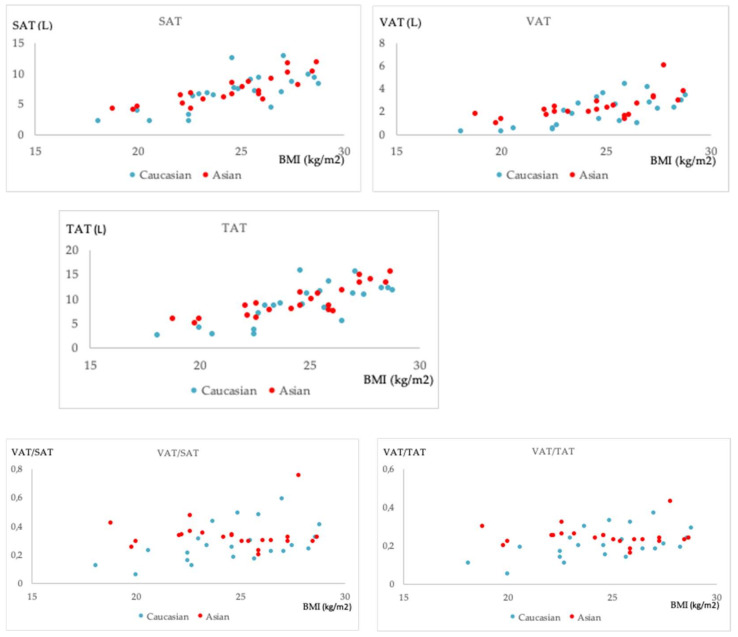
Scatter plots illustrating the relationships of fat measurements with BMI: SAT and VAT (top row: left to right), TAT (middle row) and VAT/SAT and VAT/TAT (bottom row: left to right) for the Caucasian (blue) and the Asian (red) group. Abbreviations: BMI: Body mass index; SAT: Subcutaneous adipose tissue; TAT: Total adipose tissue; VAT: Visceral adipose tissue.

The correlation coefficients (r, rho) calculated for the relationships of adipose tissue measurements with age and BMI for each group and their statistical significance are shown in Table 3. Fat measurements were independent of age in both groups. BMI was strongly correlated (r, rho > 0.6) with VAT, SAT, and TAT in both groups. Interestingly, VAT/SAT and VAT/TAT were also positively correlated with BMI in the Caucasian group (rho = 0.49 and 0.53, respectively, *p* < 0.05), but not in the Asian group (*p* > 0.05). An example of two Asian subjects with similar VAT/SAT ratio, but different BMI are shown in Figure 3. Plots of the relationships of fat measurements with BMI illustrating the aforementioned trends are shown in Figure 4. 

## 4. Discussion

In the present analysis, we compared adipose tissue volumes between groups of Asian and Caucasian postmenopausal women, matched for age and BMI, using MRI for quantification of the complete abdominal and pelvic adipose tissue. We found that absolute volumes of central adipose tissue did not significantly differ between the two groups, while the ratios of visceral to subcutaneous and visceral to total adipose tissue volumes (VAT/SAT, VAT/TAT) were higher in the Asian group. Adipose tissue volumes and age were not related in either group. Furthermore, VAT/SAT and VAT/TAT showed an increasing trend relative to BMI in the Caucasian cohort, but not in the Asian cohort.

The results of our analysis showing increased VAT/SAT and VAT/TAT ratios in the Asian compared to the Caucasian cohort are in agreement with previous studies comparing central adiposity between Asian and Caucasian groups. In a study on Japanese and Caucasian middle-aged men (40–49 years old), the Japanese group was found to have a higher VAT area and VAT/SAT ratio than their Caucasian counterparts at the same levels of obesity, measured by waist circumference [28]. It has also been reported that although East and Southeast Asians tend to have smaller SAT and VAT areas, the VAT/SAT ratio is higher in these two groups than in Caucasian, African-American, and Hispanic groups [18]. A study comparing premenopausal Asian-American with European-American women, and Asian-American with European-American men (18–44 years old) concluded that Asian-American women had a higher VAT/TAT mass ratio than European-American women, after controlling for age and total body fat mass (TFM), while such differences were not observed in men [27]. Finally, an MRI study of Japanese- and Caucasian-American postmenopausal women of similar age and BMI range showed that Japanese-American women with similar levels of adiposity had higher levels of total abdominal and visceral fat areas at the L_4_-L_5_ level, while age did not affect differences in visceral fat area and VAT/SAT area ratio between the two groups [30]. In the same study, controlling for BMI instead of TFM also resulted in a higher VAT/TAT area percentage at the L_4_-L_5_ level in the female Asian-American cohort. It should be noted that in our analysis, we did not use BMI and age as covariates, as the two groups were matched for age and BMI, with a strict one-to-one correspondence.

We did not find a correlation between age and adipose tissue measurements in the current analysis, which is inconsistent with previous studies showing an increasing visceral fat storage with age [8,9]. This discrepancy might be explained by differences in the design, cohorts, and sample sizes of studies. In the present analysis, correlations between age and fat measurements were not performed in separate BMI ranges, as was the case in [9]. In contrast to the aforementioned studies, the cohort in our analysis was not large or representative of the general population but rather consisted only of a limited sample of postmenopausal women within a small age range. Another influencing factor in our specific cohort might also be the duration of postmenopause, which was not considered in the analysis.

In the present analysis, there was a linear trend between absolute measurements of fat and BMI in both cohorts. The same was observed for the ratios VAT/SAT and VAT/TAT in the Caucasian, but not the Asian, cohort. In a study that did not include Asian subjects, BMI has been found to have a linear relationship with visceral fat accumulation in African-American, Hispanic-American, and Caucasian women, with the relationship being stronger in the Caucasian group [14]. The present finding that in the Asian group VAT/SAT and VAT/TAT ratios are not correlated with age and BMI furthermore adds to the observations of obesity-related diseases reported in Asian populations at lower BMIs compared to other racial groups [44,45]. 

The present analysis has several advantages over similar studies. Truncal adiposity in postmenopausal women of different ethnic backgrounds has already been studied [30], but in the present analysis, the participants resided in their native geographical region. This is expected to eliminate concerns raised by the effects of a modified lifestyle (e.g., an Asian population following a western lifestyle or diet) in body composition [45]. Additionally, instead of comparing two groups within a certain range of age and BMI, the present study used two strictly one-to-one age- and BMI-matched groups, in an effort to keep these parameters as constant as possible and eliminate potential biases. Finally, while most comparative analyses performed between different ethnic groups have been based on single slice measurements, our analysis is the first to do so based on a volumetric approach. As different optimal single-slice measurement sites for VAT have been suggested, depending on age, sex, and ethnicity [8], comparing volumes based on a multi-slice measurement removes the concern that the single-slice measurements are not representative of the actual VAT volume in one group. The segmentation of adipose tissue was performed semi-automatically with manual corrections, so the amount of post-processing time required was limited.

These advantages came with several limitations. Firstly, with 22 participants per group, our sample size was limited, thus results have to be interpreted with caution. Larger cohorts are needed to verify the findings of the present analysis. Second, the two groups were sampled from separate studies, and imaging took place using two different scanners. The scan protocols used were largely similar; however, slice thickness differed (Asian cohort: 2 mm, Caucasian cohort: 5 mm) and the scan time in the Asian cohort was longer, thus the images were not acquired using a breathhold sequence in the Asian cohort. Despite this, movement artifacts caused by respiratory excursion were most prominent in slices higher than the top of the liver, which was not included in the analysis. As a result, breathing during image acquisition did not seem to affect image quality in the segmented areas to the extent that limited the quality of the segmentation. Furthermore, visceral fat accumulates in the lower levels of the torso, where the lower occurrence of breathing artifacts is expected. Consequently, we can argue that even in the presence of motion artifacts in the top slices of the analyzed volume, the effect on volumetric measurements of the adipose tissue depots, especially visceral fat, is not significant. Inter- or intra-reader testing for the agreement of the manual corrections within the segmentation process was not performed due to the time-consuming nature of the manual corrections. However, in order to reach a consensus in the manual corrections, they were performed by one researcher per ethnic group who initially agreed on a common approach. For the Caucasian cohort, the number of years into postmenopause was not available while in the Singaporean cohort, only women who were at least five years postmenopausal were included. This difference should be at least partly compensated for by matching the groups by age. However, the question of whether the number of postmenopausal years influences body composition irrespective of age remains and needs to be answered in a larger study with all required information available. Finally, information such as waist and hip circumference and total body fat content was not available for the Caucasian cohort; thus, it was not considered in the statistical analysis.

In conclusion, this analysis provides insight into the differences in abdominal adipose tissue distribution between Caucasian and Asian postmenopausal women of similar age and BMI. The presented results are in agreement with previous reports of higher abdominal adiposity in Asian populations. Compared to other ethnic groups, such studies are limited in Asians, and particularly in Asian postmenopausal women. Knowledge of possible patterns of adipose tissue accumulation in distinct groups, like the ones compared in the current study, is helpful in the evaluation of disease risk and tailoring disease prevention to patient characteristics. Ethnicity should thus be considered besides sex and age when choosing the diagnostic approaches for risk evaluation and planning prevention and therapy approaches. This is particularly important in postmenopausal women, as at this stage of life, they might be particularly susceptible to cardiovascular disease and type 2 diabetes, diseases that are closely linked to visceral fat accumulation. Although further analysis is required to examine the extent to which this pattern of VAT accumulation may impact health outcomes, the results show that healthy lifestyle behaviors should be encouraged among non-obese postmenopausal women of Chinese ancestry. 

## Figures and Tables

**Table 1:** MRI sequence parameters used in both cohorts.

	Caucasian Group	Asian Group
TR	4 msec	6.55 msec
TE1/TE2	TE1 = 1.32 msec/ TE2 = 2.6 msec	TE1 = 1.35 msec/ TE2 = 2.58 msec
Flip angle	10°	9°
Field of view (FOV)	500 × 446 mm^3^	450 × 450 mm^3^
Acquisition voxel	1.5 × 2.0 × 5.0 mm^3^	1.4 × 1.4 × 2.0 mm^3^
Scan time	10 sec	116 sec

Abbreviations: TR: Repetition time, TE: Echo time.

**Table 2:** Characteristics of each group and significance value of difference between the two groups (p). “Analysed region” refers to the volume of interest, from the top of the liver to the middle of the femoral head.

	Caucasian Group (*n* = 22)	Asian Group (*n* = 22)	*p*
Normally distributed	Mean (SD, range)	Mean (SD, range)	
BMI (kg/m^2^)	24.62 (0.61, 18.1–28.8)	24.51 (0.6, 18.8–28.7)	NS
Height (m)	1.66 (0.01, 1.56–1.80)	1.56 (0.01, 1.45–1.67)	<0.01
Analyzed region length (m)	0.35 (0.01, 0.3–0.4)	0.36 (0.005, 0.32–0.40)	NS
Analyzed region length/Height	0.21 (0.003, 0.18–0.24)	0.23 (0.003, 0.21–0.25)	<0.01
SAT (L)	6.85 (0.65, 2.08–12.72)	7.13 (0.5, 3.96–11.76)	NS
TAT (L)	8.85 (0.87, 2.33–15.65)	9.49 (0.67, 4.94–15.51)	NS
Age (years)	57.15 (54–68)	58 (55–67)	NS
VAT (L)	2.11 (0.21–4.39)	2.13 (0.98–5.98)	NS
VAT/SAT ratio	0.25 (0.06–0.59)	0.32 (0.20–0.75)	0.02
VAT/TAT ratio	0.2 (0.05–0.37)	0.24 (0.16–0.43)	0.02

Abbreviations: BMI: Body mass index; SAT: Subcutaneous adipose tissue; TAT: Total adipose tissue; VAT: Visceral adipose tissue; SD: Standard deviation; NS: Not significant.

**Table 3:** Correlation results of relationships between adipose tissue measurements and age/BMI for each group.

	Caucasian Group				Asian Group			
	Age		BMI		Age		BMI	
	rho	p	r	*p*	rho	p	r/rho	*p*
**VAT (l)**	−0.12	NS	0.69	<0.01	−0.14	NS	0.67	<0.01
**SAT (l)**	−0.20	NS	0.72	<0.01	0.03	NS	0.85 ^r^	<0.01
**TAT (l)**	−0.18	NS	0.75	<0.01	−0.02	NS	0.85 ^r^	<0.01
**VAT/SAT**	−0.29	NS	0.49	0.02	−0.21	NS	−0.25	NS
**VAT/TAT**	−0.03	NS	0.53	0.01	−0.23	NS	−0.23	NS

Abbreviations: BMI: Body mass index; SAT: Subcutaneous adipose tissue; TAT: Total adipose tissue; VAT: Visceral adipose tissue; r: Pearson correlation coefficient; rho: Spearman’s rho; NS: Not significant.

## Data Availability

The processed data presented in this study are available on request from the corresponding author. The original imaging data are not publicly available due to privacy reasons given the sensitivity of patient data.
